# Optogenetic Analysis of Depolarization-Dependent Glucagonlike Peptide-1 Release

**DOI:** 10.1210/en.2017-00434

**Published:** 2017-07-28

**Authors:** Catalin Chimerel, Cristian Riccio, Keir Murison, Fiona M. Gribble, Frank Reimann

**Affiliations:** 1 Metabolic Research Laboratories and Medical Research Council (MRC) Metabolic Diseases Unit, Wellcome Trust–MRC Institute of Metabolic Science, Addenbrooke’s Hospital, University of Cambridge, Cambridge CB2 0QQ, United Kingdom

## Abstract

Incretin hormones play an important role in the regulation of food intake and glucose homeostasis. Glucagonlike peptide-1 (GLP-1)–secreting cells have been demonstrated to be electrically excitable and to fire action potentials (APs) with increased frequency in response to nutrient exposure. However, nutrients can also be metabolized or activate G-protein–coupled receptors, thus potentially stimulating GLP-1 secretion independent of their effects on the plasma membrane potential. Here we used channelrhodopsins to manipulate the membrane potential of GLUTag cells, a well-established model of GLP-1–secreting enteroendocrine L cells. Using channelrhodopsins with fast or slow on/off kinetics (CheTA and SSFO, respectively), we found that trains of light pulses could trigger APs and calcium elevation in GLUTag cells stably expressing either CheTA or SSFO. Tetrodotoxin reduced light-triggered AP frequency but did not impair calcium responses, whereas further addition of the calcium-channel blockers nifedipine and *ω*-conotoxin GVIA abolished both APs and calcium transients. Light pulse trains did not trigger GLP-1 secretion from CheTA-expressing cells under basal conditions but were an effective stimulus when cyclic adenosine monophosphate (cAMP) concentrations were elevated by forskolin plus 3-isobutyl 1-methylxanthine. In SSFO-expressing cells, light-stimulated GLP-1 release was observed at resting and elevated cAMP concentrations and was blocked by nifedipine plus *ω*-conotoxin GVIA but not tetrodotoxin. We conclude that cAMP elevation or cumulative membrane depolarization triggered by SSFO enhances the efficiency of light-triggered action potential firing, voltage-gated calcium entry, and GLP-1 secretion.

## Teaser

This optogenetic study suggests that a small maintained membrane depolarization is needed to achieve optimal action potential frequencies, voltage-gated calcium entry, and GLP-1 secretion.

Enteroendocrine cells (EECs) are hormone-secreting cells localized in the gut epithelium, many of which are directly activated upon exposure to nutrients. The gut hormones secreted from EECs modulate multiple physiologic responses, including gastrointestinal motility, food intake, energy expenditure, and glucose homeostasis (1). Sensing of the luminal contents by EECs relies on molecular recognition by G-protein–coupled receptors and electrogenic nutrient cotransporters (2).

Particularly in enteroendocrine L cells, a subpopulation of EECs producing glucagonlike peptide-1 (GLP-1), there is solid evidence for the expression of voltage-gated sodium and calcium channels enabling these cells to fire action potentials (3, 4). Elevation of extracellular glucose has been demonstrated to depolarize and increase the frequency of action potentials (APs) in both the well-established L-cell model line GLUTag (5, 6) and primary L cells in mixed epithelial cultures (7). The sodium-coupled glucose cotransporter, SGLT1, is critical for this effect, with small transporter associated inward Na^+^ currents depolarizing the membrane potential of L cells (6, 8, 9). Further evidence for the importance of electrogenic transport for L-cell nutrient sensing comes from the attenuation of GLP-1 secretion in response to dipeptides/tripeptides by pharmacologic inhibition or knockout of the proton-coupled peptide transporter, PEPT1 (10). Methyl-aminoisobutyric acid, a nonmetabolized substrate for sodium-coupled system A amino acid transport, elevated Ca^2+^ in GLUTag (11) and primary L cells (12) and glutamine-triggered GLP-1 secretion was sensitive to tetrodotoxin (TTX), an inhibitor of voltage-gated sodium channels (3)

A consensus model for GLP-1 secretion has thus been developed, in which electrogenic uptake of nutrients results in a small depolarization from the resting membrane potential, sufficient to trigger action potentials and activation of voltage-gated calcium channels. The increase in cytosolic Ca^2+^ subsequently increases the exocytosis of GLP-1–loaded vesicles (13). Mathematical modeling with experimentally derived cell parameters further confirmed the validity of this model (14, 15).

Measurements of GLP-1 secretion stimulated directly by membrane depolarization, without the use of pharmacologic agents that might additionally enhance secretion through alternative targets, have to our knowledge not been reported. Elevation of extracellular K^+^ has, however, been reported to stimulate neuronal transmitter release independently of its depolarizing action (16), and sulfonylureas used to close adenosine triphosphate–sensitive potassium channels have been shown to also affect the cyclic adenosine monophosphate (cAMP) sensor EPAC2 (17). In support of the idea that electrophysiologic stimuli are sufficient to trigger GLP-1 secretion, depolarization of the membrane potential to activate voltage-gated Ca^2+^ entry was associated with an increase of membrane capacitance, a measure of vesicular secretion, in GLUTag cells in perforated-patch-clamp whole-cell mode (18), but this single cell technique is not suitable to measure hormone secretion itself. Here we used the GLUTag cell line as a model of enteroendocrine L cells and optogenetic probes genetically expressed in the plasma membrane of GLUTag cells to control the membrane potential. Stimulation protocols using light pulses confirmed that membrane depolarization was sufficient to induce GLP-1 secretion.

## Materials and Methods

### Generation of stable cell lines

Plasmids expressing the channelrhodopsin variants SSFO [hChR2(C128S/D156A)] and CheTA [hChR2(E123T/H134R)] were purchased from Addgene (catalog no. 35504 and catalog no. 26968, respectively). Because these were DIO-Cre reporter constructs for the generation of adeno-associated viruses, the SSFO-mCherry and CheTA-EYFP coding sequences were isolated through a *Nhe*I/BsrGI digest and cloned into pcDNA3.1(-), which contained a 3′ BsrGI site due to an mCherry containing cassette.

### GLUTag-C

GLUTag cells were transfected with the CheTA plasmid by using Lipofectamine 2000 (ThermoFisher Scientific) as a transfection reagent. Stably transfected cells were further selected by using 0.5 mg/mL Geneticin Sulfate (Santa Cruz Biotechnology). From this cell population, a single cell colony with good GFP-CheTA expression was isolated and used in the subsequent experiments.

### GLUTag-S

GLUTag cells were transfected with the SSFO plasmid by using Lipofectamine 2000 as a transfection reagent. Stably transfected cells were further selected by using 0.5 mg/mL Geneticin Sulfate, and initially the brightest cells of the population (top 5%) were subcloned by using fluorescence-assisted cell sorting. GLUTag-C and GLUTag-S lines were established from the parent GLUTag clone in the Reimann/Gribble laboratory (<25 passages); once subclones had been established, these were used experimentally for up to 20 additional passages.

### Optogenetic setup

**Figure 1.**
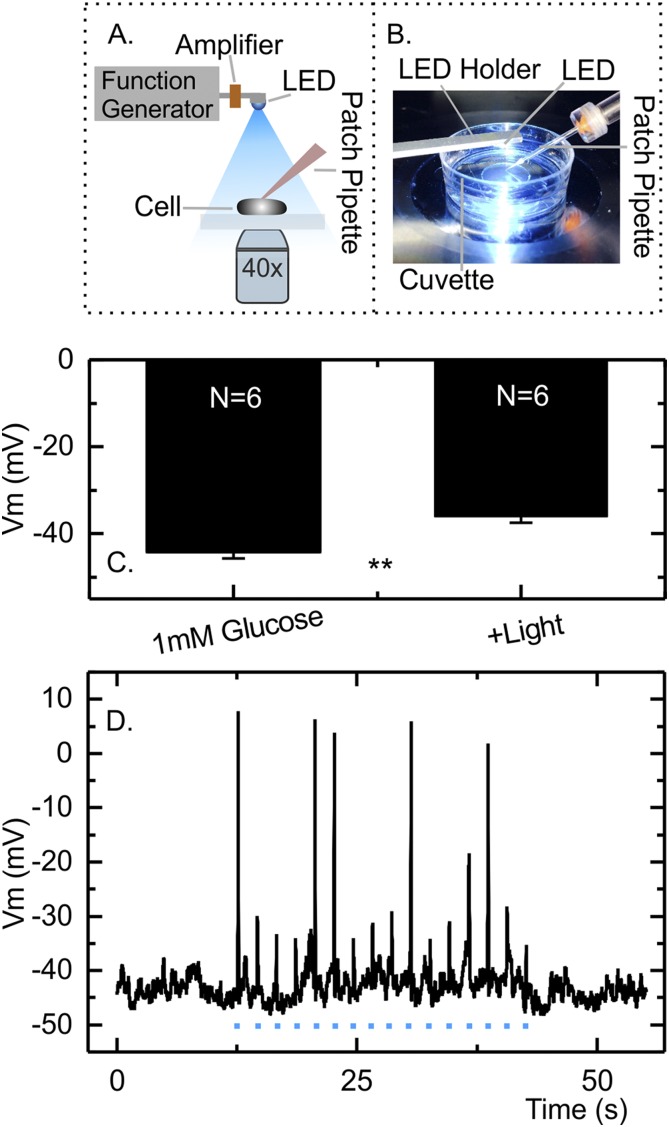
Membrane depolarization in response to light in the GLUTag-C cell line. (A) Schematic representation of the custom-built optogenetic setup. (B) Photograph of the cell-containing glass-bottom dish illuminated with LED light. (C) Mean ± standard error of the mean resting membrane potential (Vm) in the presence and absence of continuous illumination (n = 6 cells). **P < 0.01 by paired Student *t* test. (D) Membrane potential of an example GLUTag-C cell recorded in perforated patch whole-cell current clamp stimulated with light pulses of 25 ms in duration, ignited from the xenon arc lamp (stimulations marked with a blue dot in the figure). Some, but not all, light pulses triggered action potentials. Two of the six GLUTag-C cells studied in this way fired light pulse stimulated action potentials.

The light pulses used to achieve optogenetic control were emitted from a high-power single-color LED with a peak wavelength centered between 460 nm and 485 nm and a luminous intensity of 40 lm when ran at 350 mA (catalog no. LXML-PB01-0040; Lumileds). The illumination of the LED was computer controlled. pCLAMP 10 software (Molecular Devices) coupled with the digitizer Digidata 1440A (Molecular Devices) was used to generate fast voltage changes between 0 and 3 V. The power of the digitizer was amplified with a Rigol PA1011–10 W power amplifier and used to light the LED at different frequencies (1 Hz, 2 Hz, and 4 Hz) and for different durations (>1.25 ms). Using custom-built printed circuit boards (Supplemental Fig. 1) this system was easily integrated into the microscope of the electrophysiology setup (Fig. 1A and 1B) and the secretion assay (Supplemental Fig. 1). For some experiments, illumination was achieved through a 75-W xenon arc lamp and a monochromator (Cairn Research) controlled by MetaFluor software (Universal Imaging) on an Olympus IX71 microscope. Optogenetic stimulation form the LED or the xenon arc lamp resulted in similar activation of the optogenetic probes, and simultaneous illumination with both devices did not result in greater Ca^2+^ elevation, suggesting maximal stimulation in response to either alone.

### Electrophysiology

GLUTag cells were patch clamped and monitored as previously described (4). The bath solution was a standard saline buffer (4.5 mM KCl, 138 mM NaCl, 2.6 mM CaCl_2_, 4.2 mM NaHCO_3_, 1.2 mM H_2_NaPO_4_, 1.2 mM MgCl_2_, and 10 mM HEPES [pH 7.4 with NaOH]) supplemented with 0.1 or 1 mM glucose. All experiments were performed in perforated patch by using an intracellular pipette solution composed of 76 mM K_2_SO_4_, 10 mM KCl, 10 mM NaCl, 55 mM sucrose, 10 mM HEPES, and 1 mM MgCl_2_ (pH 7.2 with KOH). The patch electrode was first tip-filled with pipette solution before being back-filled with 1 to 2 µg/mL amphotericin (diluted from a 1-mg/mL dimethyl sulfoxide stock) in pipette solution. The tip of the microelectrode was fire polished by using a microforge (MF-830; Narishige) and had a resistance of 2.5 to 4 MΩ when filled with pipette solution. Membrane potentials were recorded in IClamp Mode with an Axopatch 200B amplifier (Molecular Devices) through a Digidata 1440A digitizer (Molecular Devices) and processed with pCLAMP 10.3 software (Molecular Devices). Data were filtered at 10 kHz by using a low-pass Bessel filter and sampled at 25 kHz. The optogenetic stimulation was triggered simultaneously with the voltage recordings using specific protocols generated in pCLAMP 10.3 (Molecular Devices). For all microscopy experiments (patch clamping and calcium imaging), the LED was supported on a custom-built printed circuit board in the form of a cantilever at approximately 1 cm from the cells when stimulating the optogenetic probes (Fig. 1B). For experiments with the slow deactivating channelrhodopsin, SSFO, for both electrophysiology studies and calcium imaging studies the microscope equipped with the xenon arc lamp and the monochromator was used to generate 590 nm light, which rapidly deactivates SSFO.

### Calcium imaging

GLUTag cells were plated on matrigel-coated 35-mm glass-bottom dishes, 1 to 2 days before use and were loaded with Fura-2-acetoxymethyl ester (Fura-2-AM; Invitrogen). The dish was mounted in a perfusion chamber on an Olympus IX71 microscope with a ×40 oil-immersion objective and imaged by using an Orca-ER charge-coupled device camera. A 75-W xenon arc lamp and a monochromator (Cairn Research) controlled by MetaFluor software were used to alternately excite the dye at 340 and 380 nm. The Fura-2-AM fluorescence was measured at >510 nm. The integration time for the 340- and 380-nm images were each 50 ms long and were consecutively fired every 4 seconds. Optogenetic excitation through the LED (or occasionally through the monochromator) was intercalated between the Fura-2 excitation without any overlap, achieved by synchronizing the MetaFluor and pCLAMP software.

### GLP-1 secretion

GLUTag-C or GLUTag-S cells were plated in white opaque 24-well plates (CulturPlate-24, 6005168; PerkinElmer) and cultured overnight. On the day of the experiment, wells were washed twice with 400 μL standard saline buffer supplemented with 0.1 mM glucose and 0.1% (weight-to-volume ratio) bovine serum albumin. The plate was covered with a printed circuit board, which positioned a single LED above each of the 24 wells, enabling application of specific light pulses. To minimize light crosstalk between wells, we used a three-dimensionally printed opaque block, which fit the grooves of the plate (Supplemental Fig. 1). GLP-1 content of the supernatants was assessed by immunoassay using a total GLP-1 assay (Mesoscale Discovery) in light-stimulated and dark-treated wells on the same plate.

## Results

To control the membrane potential of GLUTag cells independently of the application of pharmacologic agents, we initially established a cell line (GLUTag-C) stably expressing the channelrhodopsin variant CheTA. CheTA has fast on/off kinetics, which allow the precise temporal control of short-lived depolarizations (19). To control the optogenetic probe, a custom-built illumination setup was developed as described in the Materials and Methods section, and light pulses of defined duration and specified frequencies were generated to activate the channelrhodopsin. Using the perforated whole-cell patch-clamp configuration, we confirmed that continuous illumination resulted in a small but significant depolarization of GLUTag-C cells from −44.2 ± 1.5 mV to −36.1 ± 1.4 mV (both in 1 mM glucose) (Fig. 1C) and increased action potential firing (Supplemental Fig. 2). Shorter light pulses of 25-ms duration triggered action potentials in a proportion of cells, although not in response to every pulse in every cell, without significantly affecting the interpulse resting membrane potential (Fig. 1D).

**Figure 2.**
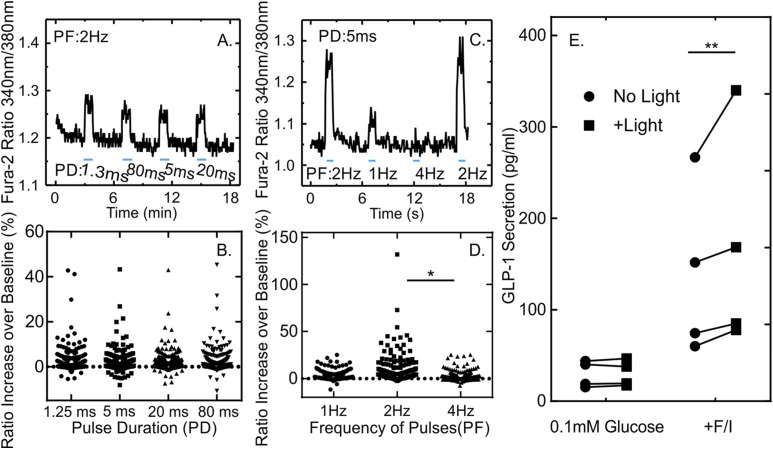
Intracellular calcium changes and GLP-1 secretion levels in response to light pulses in GLUTag-C cells. (A) Fura-2 340/380 ratio of a GLUTag-C cell stimulated with light pulses of different duration. Light trains each comprised 100 individual light pulses of variable duration (1.3 to 80 ms), applied at 2 Hz with an LED as indicated by the lines. (B) Scatter plot of the percentage increase of the Fura-2 340/380 ratio over baseline in response to light pulses of different duration applied at 2 Hz to GLUTag-C cells in 1 mM glucose as in part (A). All monitored cells (n = 256), regardless of their responsiveness, are shown. No significant difference was detectable between conditions, as assessed by analysis of variance (ANOVA) comparing the mean responses calculated for each independent experiment (n = 8) (C) Fura-2 340/380 ratio of a GLUTag-C cell in response to light pulses applied at different frequencies. Each pulse train was 50 seconds long and was composed of pulses with a duration of 5 ms, applied at frequencies ranging from 1 Hz to 4 Hz as indicated by the bars. (D) Scatterplot of the percentage increase of the Fura-2 340/380 ratio over baseline in response to light pulses of 5 ms in duration applied to GLUTag-C cells in 1 mM glucose at 1 to 4 Hz as in part (C). All monitored cells (n = 208), regardless of their responsiveness, are shown. Results were compared using one-way ANOVA followed by Bonferroni multiple comparison test using the mean responses calculated from all cells in each independent experiment (n = 6). *P < 0.05. When frequency responses were pairwise compared for all cells individually, responses to 2 Hz were highly significantly different from both 1 Hz and 4 Hz (*P* < 0.001 for both comparisons). (E) GLP-1 secretion from GLUTag-C cells in the presence of 0.1 mM glucose or 0.1 mM glucose + forskolin/IBMX (10 µM each) with and without light stimulation. Light pulses of 5 ms in duration were applied at 2 Hz for 20 minutes (equal to 2400 LED pulses). Data are presented as scatterplot showing secretory responses in the absence (circles) or presence (squares) of light. Each point represents the mean of two to four wells measured in parallel, and results from the same experiment are linked by lines. Significance was tested with a two-way repeated-measures ANOVA and Bonferroni multiple comparisons test performed on log-transformed data (n = 4). **P < 0.01.

To further characterize how short light pulses affected GLUTag-C cells, we monitored cytosolic Ca^2+^ dynamics after loading cells with Fura-2-AM. Cytosolic Ca^2+^ dynamics can be considered as a proxy of the activation of Ca^2+^-permeable channelrhodopsins and/or activation of voltage-gated Ca^2+^ channels, allowing the simultaneous monitoring of many more cells than is possible with electrophysiologic recordings and avoiding possible changes in the membrane potential due to the observation method. Using short light pulses of 5 ms in duration, we observed that a maximal Ca^2+^ response was achieved when pulses were applied at 2 Hz, with smaller Ca^2+^ elevations at lower and higher frequencies (Fig. 2C and 2D) Interestingly, variation of the light-pulse duration between 1.2 and 80 ms had no significant effect on the Ca^2+^ response at 2 Hz (Fig. 2A and 2B).

To determine whether optogenetic-induced calcium changes translated into GLP-1 secretion, we measured GLP-1 release from GLUTag-C cells in response to light (Fig. 2E). GLP-1 secretion in 0.1 mM glucose was not affected by a 20-minute pulse train of 5-ms pulses at 2 Hz (Fig. 2E). GLP-1 secretion was, however, significantly increased by light in the additional presence of forskolin and 3-isobutyl 1-methylxanthine (IBMX; 21% increase) (Fig. 2E). We concluded that the elevation of cytosolic Ca^2+^ observed in some GLUTag-C cells at basal cAMP levels was insufficient to support a robust elevation of GLP-1 release whereas cAMP elevation supported light-triggered secretion.

In previous work we observed strong stimulation of GLP-1 secretion from GLUTag cells by glucose, even when action potentials were blocked by TTX (4). At the time we concluded that preserved depolarization under these conditions was sufficient to activate voltage-gated Ca^2+^ channels, triggering Ca^2+^ elevation and GLP-1 secretion. On the basis of these findings, we hypothesized that a prolonged depolarization of GLUTag-cells, as observable under continuous illumination of CheTA (Fig. 1C and Supplemental Fig. 2), might be a better stimulus for GLP-1 secretion than individual short light pulses. Unfortunately, continuous illumination resulted in nonnegligible heating of the incubation chamber during the longer incubation times required for secretion experiments. To enable long-lasting depolarization in response to relatively short light pulses, we decided to use SSFO, which has deactivation times in the range of 30 minutes but can be rapidly deactivated by exposure to 590 nm or ultraviolet light (20, 21). We established another stable cell line, GLUTag-S, expressing SSFO.

**Figure 3.**
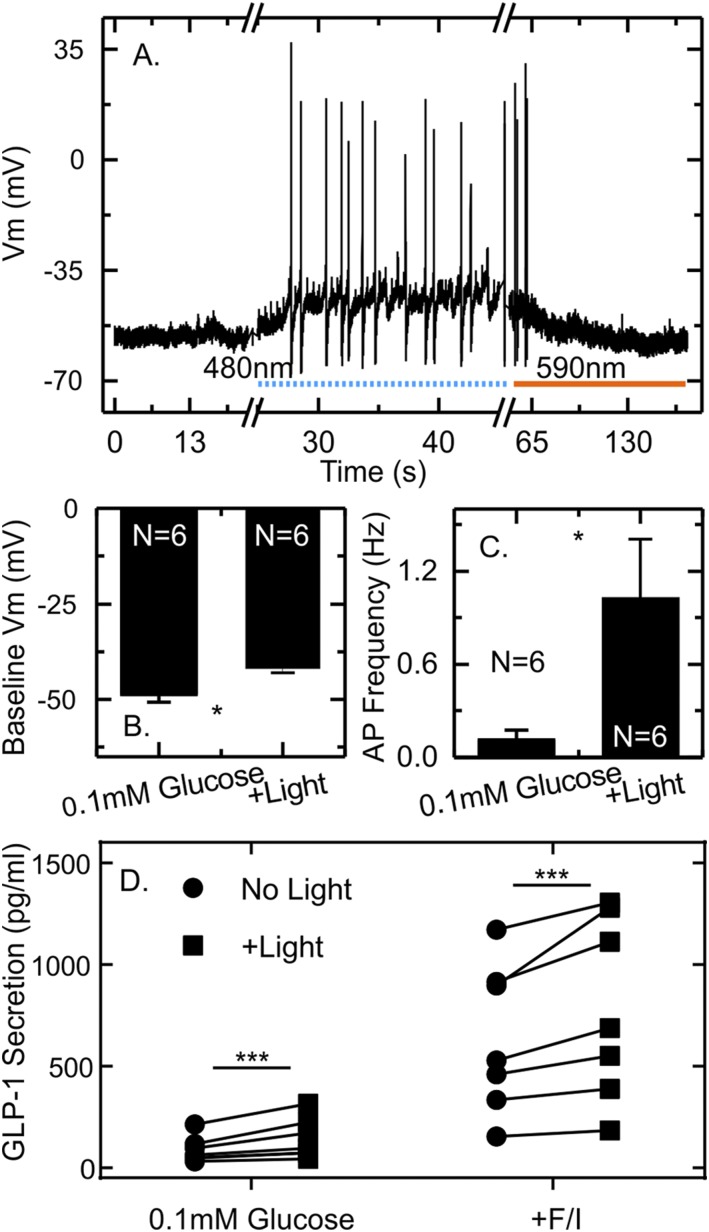
Membrane depolarization and GLP-1 secretion in response to light pulses in GLUTag-S cells. (A) Membrane potential of an example GLUTag-S cell recorded in perforated patch whole cell current clamp in 0.1 mM glucose in response to a train of 5-ms LED light pulses applied at 2 Hz. Each light pulse is marked by a dot in the figure. Deactivation of SSFO channelrhodopsin was achieved by illumination at 590 nm as indicated by the bar. (B) Mean ± standard error of the mean resting membrane potential (Vm) of GLUTag-S cells in the presence and absence of pulsed illumination as in part (A) (n = 6 cells). (C) Mean ± standard error of the mean action potential frequency in GLUTag-S cells in the presence and absence of light pulses as in part (A) (n = 6 cells). (D) GLP-1 secretion from GLUTag-S cells in the presence of 0.1 mM glucose, with or without addition of forskolin/IBMX (10 μM each), in the presence and absence of pulsed illumination. Pulses 5 ms in length were applied at 2 Hz for 20 minutes (equal to 2400 LED pulses). Data are presented as scatterplot showing secretory responses in the absence (circles) or presence (squares) of light. Each point represents the mean of three to four wells measured in parallel, and results from the same experiment are linked by lines. Significance was tested with a two-way repeated-measures analysis of variance and Bonferroni multiple comparisons test, performed on log-transformed data (n = 4). ***P < 0.001.

Confirming the action of SSFO, the membrane potential of GLUTag-S cells recorded in 0.1 mM glucose was depolarized from −48.6 ± 2.1 mV to −42.0 ± 1.0 mV in response to short light pulses and returned to resting levels when SSFO was deactivated by illumination at 590 nm (Fig. 3A and 3B). Consistent with the long deactivation time of SSFO, the depolarization was long lived and accompanied by spontaneous firing of action potentials (Fig. 3A), which increased in frequency from a basal rate of 0.1 Hz to a light-stimulated rate of 1 Hz (Fig. 3C). Notably, the light induced activation of SSFO increased GLP-1 secretion significantly both in the presence of 0.1 mM glucose and 0.1 mM glucose plus forskolin and IBMX (Fig. 3D). A 20-minute light train of 5-ms pulses at 2 Hz increased GLP-1 secretion by 58% in 0.1 mM glucose and by 23% in 0.1 mM glucose plus forskolin and IBMX.

**Figure 4.**
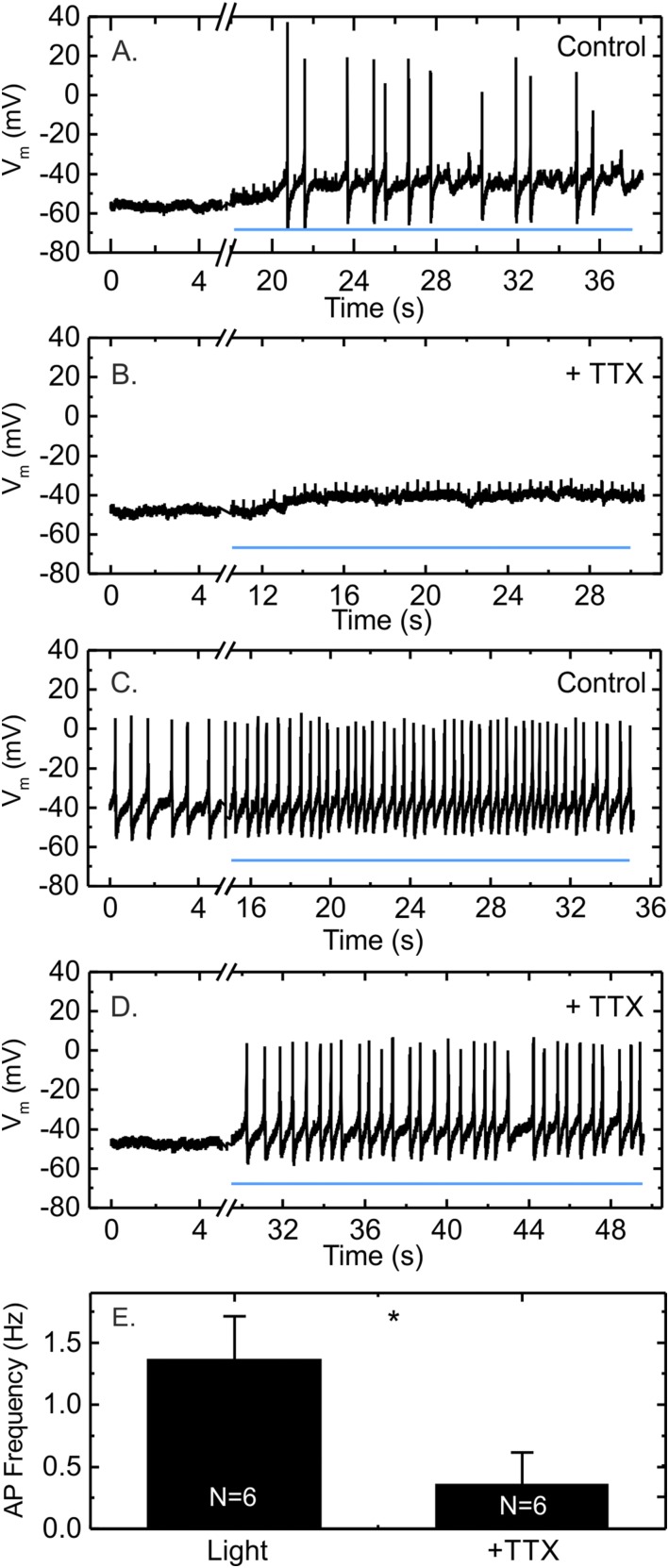
The effect of TTX on light-induced action potential firing in GLUTag-S cells. (A, B) Example trace of a GLUTag-S cell recorded in perforated whole-cell current clamp in 0.1 mM glucose in which TTX (3 μM) completely inhibited light-induced action potential firing but did not prevent cumulative depolarization. (C, D) Example trace of a cell recorded as in (A, B), in which light-triggered action potentials were still observed in the presence of TTX. The continuous line marks the train of 5-ms LED light pulses fired at 2Hz. (E) Average action potential frequency during light stimulation in the absence and the presence of TTX (3 µM) measured for n = 6 cells. The data are presented as mean ± standard error of the mean. *P < 0.05 by paired Student *t* test. Vm, resting membrane potential.

We next investigated the role of voltage-gated ion conductances in light-stimulated GLP-1 secretion from GLUTag-S cells. In perforated patch-clamp recordings, TTX completely blocked action potential firing in three of six cells investigated, whereas a net depolarization in response to light was still observable (Fig. 4A and 4B). In the other three cells, light still triggered action potentials in the presence of TTX but at a reduced frequency compared with the light-stimulated activity before TTX application (Fig. 4C and 4D). Overall, application of TTX significantly reduced the action potential frequency triggered by light (Fig. 4E).

**Figure 5.**
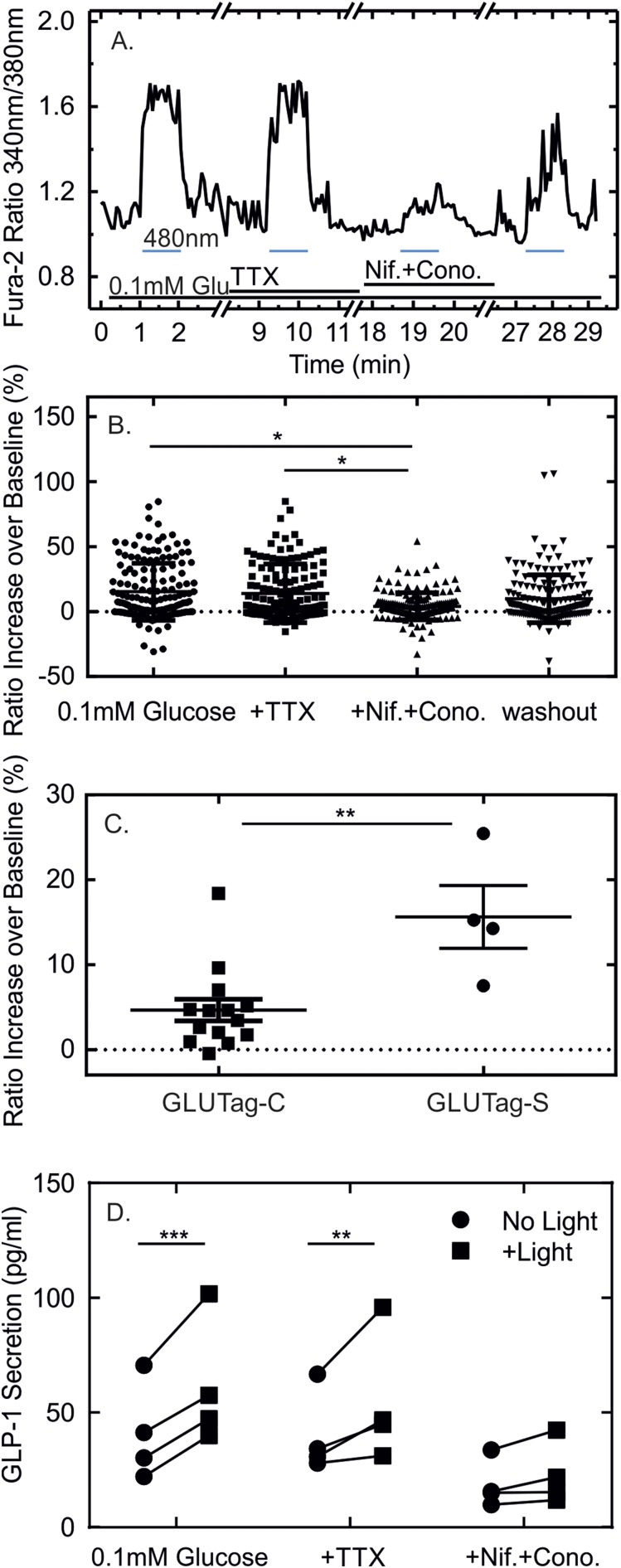
The effects of ion channel blockers on light-induced intracellular calcium responses and GLP-1 secretion in GLUTag-S cells. (A) Fura-2 340/380 ratio of a GLUTag-S cell, in response to 100 light pulses of 5 ms in duration applied at 2 Hz as indicated by the lines. Initial responses were recorded in 0.1 mM glucose in saline (basal) and subsequent responses in the additional presence of TTX (3 μM) without or with *ω*-conotoxin-GVIA (Cono; 1 μM) + nifedipine (Nif; 5 μM), or after washout of the ion-channel inhibitors as indicated. (B) Scatterplot of the percentage increase of the Fura-2 340/380 ratio over baseline in response to light pulses in the presence and absence of ion-channel inhibitors in GLUTag-S cells as in part (A). All monitored cells (n = 159), regardless of their responsiveness are shown. Results were compared by using one-way analysis of variance (ANOVA) followed by Bonferroni multiple comparison test using the mean responses calculated from all cells in each independent experiment (n = 4). *P < 0.05. (C) Comparison of Fura2 340/380 responses to 100 light pulses of 5 ms in duration applied at 2 Hz in GLUTag-C and GLUTag-S cells. Data are presented as scatterplot of the means calculated from all cells in each independent experiment shown in Fig. 2 (GLUTag-C, n = 14) and Fig. 3 (GLUTag-S, n = 4) and were compared by Student *t* test. **P < 0.01. (D) GLP-1 secretion from GLUTag-S cells in response to light pulses in the presence and absence of ion channels blockers at concentrations as in part (A). Each light pulse was 5 ms in duration, and the train consisted of 2400 pulses fired at 2 Hz (equal to 20 minutes of stimulation). Data are presented as scatterplots showing secretory responses in the absence (circles) or presence (squares) of light. Each point represents the mean of three to four wells measured in parallel, and results from the same experiment are linked by lines. Significance was tested by two-way repeated-measures ANOVA and Bonferroni multiple comparisons test, performed on log-transformed data (n = 4). **P < 0.01; ***P < 0.001.

Consistent with the initiation of light-induced APs, the optogenetic-induced depolarization also increased intracellular calcium levels in GLUTag-S cells in response to 5-ms light pulses at 2 Hz (Fig. 5A and 5B). The rapid decrease in calcium observed after the cessation of light pulses in these experiments was largely attributable to the deactivation of SSFO by the ultraviolet illumination used to excite Fura-2; additional exposure to 590 nm was used between pulse trains to ensure complete SSFO deactivation between the test conditions. Mean calcium responses to 2-Hz 5-ms light pulse trains across all cells examined were higher in GLUTag-S than GLUTag-C cells (Fig. 5C). The addition of TTX did not, however, impair the light-triggered rise in cytosolic calcium (Fig. 5A and 5B), consistent with the ability of GLUTag-S cells to fire Ca^2+^ channel–dependent action potentials (Supplemental Fig. 3). By contrast, addition of the voltage-gated L-type and N-type Ca^2+^-channel blockers nifedipine (5 µM) and *ω*-conotoxin GVIA (1 µM) significantly reduced the light-induced increase in cytosolic calcium (Fig. 5A and 5B). Light-induced GLP-1 secretion from GLUTag-S cells was similarly not affected by TTX, whereas the addition of 5 µM nifedipine and 1 µM *ω*-conotoxin GVIA reduced GLP-1 secretion in both the absence and presence of light (Fig. 5D).

## Discussion

Previous reports correlated changes in L-cell electrical activity in response to nutrients with stimulation of hormone release; the optogenetic control used here confirms depolarization of the plasma membrane to be a sufficient stimulus for GLP-1 secretion in GLUTag cells. When SSFO was expressed in GLUTag cells (GLUTag-S), we observed a cumulative membrane depolarization in response to repeated brief light pulses, action potential firing, elevated cytosolic Ca^2+^, and a corresponding significant increase in GLP-1 release.

The light-sensitive stimulation of GLUTag-S cells depended on the recruitment of voltage-gated ion currents. Both Ca^2+^ elevations and secretion were blocked by the addition of pharmacologic inhibitors of L- and N-type Ca^2+^-channels, which have been previously reported to underlie GLP-1 secretion from GLUTag cells (4). This excludes the possibility that Ca^2+^ entry through SSFO itself is sufficient to stimulate GLP-1 secretion above basal levels. Although TTX reduced the light-triggered action potential firing frequency, it did not significantly reduce GLP-1 secretion, mirroring our previous finding that TTX did not block glucose-stimulated secretion from GLUTag cells (4). The observation of action potentials in the presence of TTX differs from our previous report in which TTX abolished electrical activity in all six GLUTag cells investigated at the time (4) but is similar to what we observed subsequently in primary L cells (3). It thus appears that voltage-gated sodium channels increase the likelihood that GLP-1–secreting cells fire action potentials in response to membrane depolarization but are redundant for secretion, provided the stimulus overcomes the threshold for activating voltage-gated Ca^2+^ channels. The majority of calcium entry responsible for GLP-1 secretion is likely carried by calcium-dependent action potentials.

Interestingly, although we achieved some elevation in cytosolic Ca^2+^ with intermittent stimulation of CheTA, which has a much faster deactivation time constant compared with SSFO (5 ms and 30 minutes, respectively) (19, 20), this did not translate into significant stimulation of GLP-1 secretion under basal conditions. A simple possibility is that insufficient numbers of cells responded to support a significant increase of GLP-1 secretion measured over the whole well population. For individual cells, an increased Ca^2+^ response upon increasing the pulse frequency from 1 to 2 Hz was expected, given that a corresponding increase in action potential frequency should build up intracellular calcium levels. This simple expected relationship did not, however, hold when the stimulation frequency was further increased to 4 Hz. Although at least some CheTA-transfected GLUTag cells were able to fire higher-frequency APs triggered by faster stimulation protocols, we noted that the action potential amplitude decreased at higher frequencies, likely resulting in less Ca^2+^ entry (Supplemental Fig. 4 and Fig. 2). Interestingly, this corresponds with the relatively slow firing frequencies previously observed in GLUTag cells and primary L cells under stimulated conditions, which were also in the range of 1 to 2 Hz. By contrast, at 2 Hz there was little dependence of Ca^2+^ on the light-pulse duration between 1.3 and 80 ms. This suggests that the short-duration pulses were themselves sufficient to trigger APs in a proportion of cells but that longer pulse durations of up to 80 ms did not support firing of additional APs. By contrast, in GLUTag-S cells, repeated light pulses resulted in a sustained depolarization and action potential initiation that was independent of light pulse timing but in accordance with the level of membrane depolarization (Fig. 3). It seems likely that the prolonged depolarization observed in GLUTag-S cells was responsible for driving the larger Ca^2+^ responses and higher GLP-1 secretory rates. This relatively small cumulative depolarization appears to be sufficient to enable spontaneous superimposed fluctuations of the membrane potential to cross the action potential threshold.

The ability of CheTA to support GLP-1 secretion in conditions of elevated cAMP supports this idea, as we previously reported that raised cAMP depolarizes the membrane and increases the excitability in GLUTag cells (22), which would increase the responsiveness to the cells to brief CheTA-driven depolarizations. A caveat of this interpretation is that we worked with subcell lines, which might have altered secretory properties unrelated to the expressed channelrhodopsin variants. However, the fact that we achieved similar cumulative depolarization in the GLUTag-C line under constant light and the GLUTag-S line with light pulses makes the trivial explanation that different expression levels of the channelrhodopsin underlie the different responsiveness unlikely.

In conclusion, we have demonstrated that light-generated membrane depolarization in GLUTag cells translates into an increased intracellular calcium level, likely carried by calcium-dependent action potentials, in turn enhancing GLP-1 secretion. In addition, we have shown that TTX-sensitive voltage-gated sodium channels are not critical for hormone secretion from GLUTag cells because calcium elevation and action potentials were observed even when the sodium channels were blocked—similar to our observations of L cells in primary culture (3). Finally, our results suggest that step function opsins, which allow a sustained depolarization in response to intermittent light pulses, are a better alternative to channelrhodopsins with fast on/off kinetics for the control of enteroendocrine hormone secretion.
