# Supplementary material for: Optogenetic Analysis of Depolarization-Dependent Glucagonlike Peptide-1 Release
Source: Endocrinology. 2017 Jul 28;158(10):3426–34. doi: 10.1210/en.2017-00434 (PMC5659701; doi:10.1210/en.2017-00434)
Supplement: Supplementary file 1 [file en.2017-00434.sf1.DOCX]

Supplementary Figures:


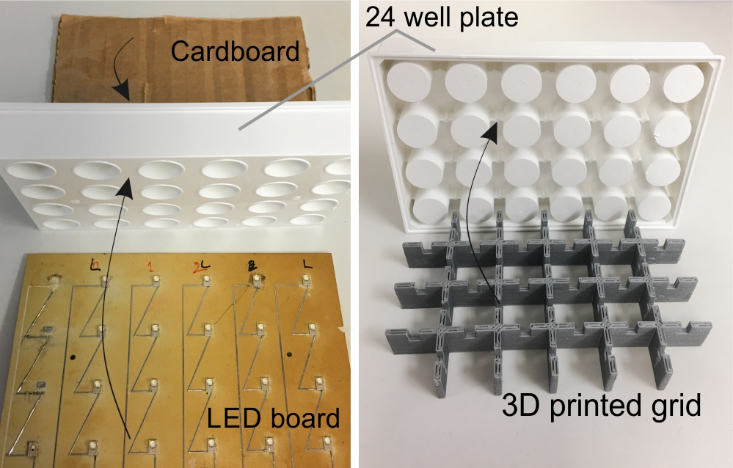


Figure S1. Photos of the 24 well plate ready to be assembled for the optogenetic measurements. The LED board is sandwiched with the 24 well plate, an opaque 3D printed block and a cardboard plate to avoid any light crosstalk between wells. The arrows indicate the direction of the assembly.


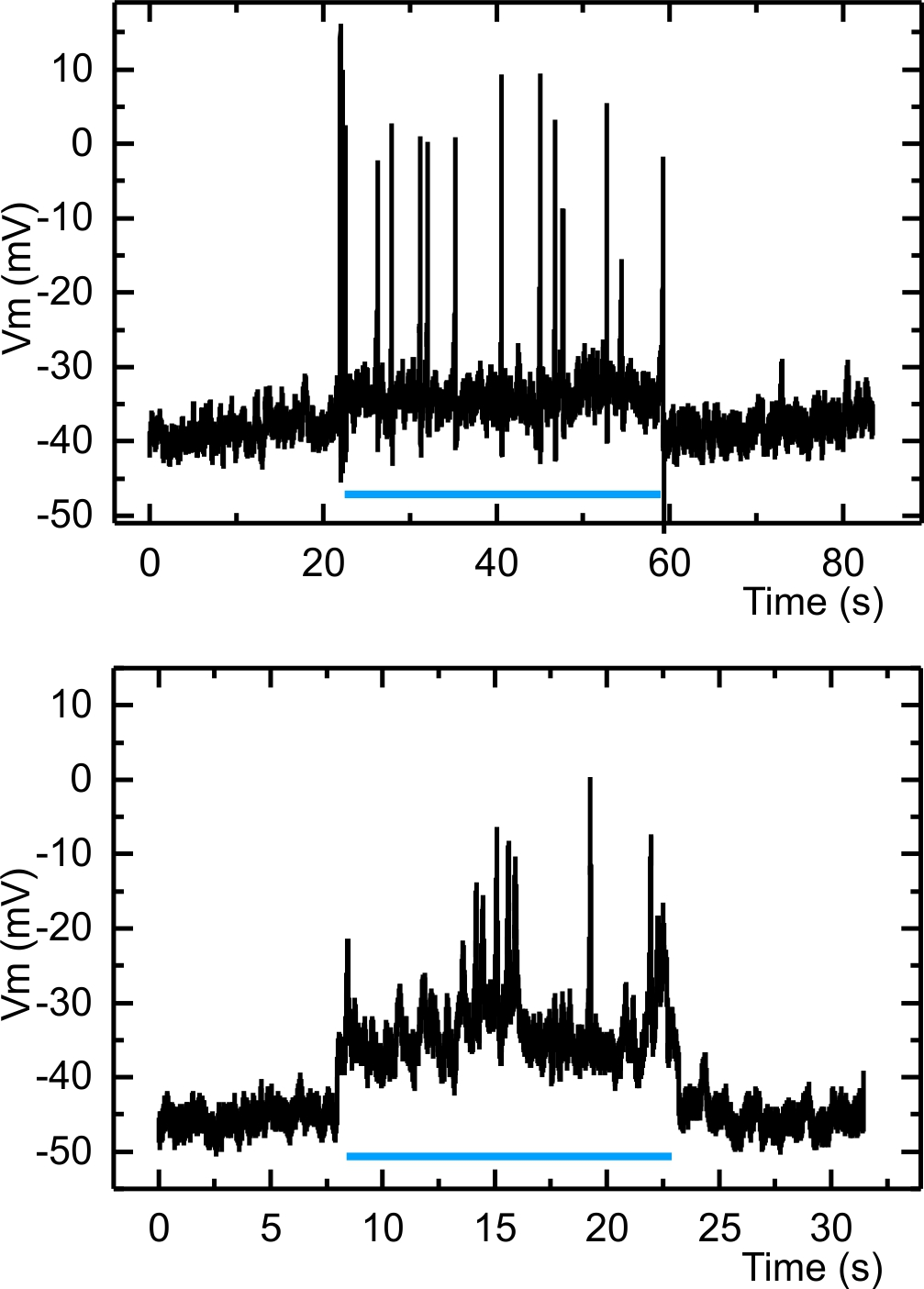


Figure S2. Depolarisation and increase in action potential firing rate in GLUTag-C cells in response to continuous light application. GLUTag-C cells in 1 mM glucose in perforated whole-cell clamp were exposed to continuous light as indicated by the blue bar.

 
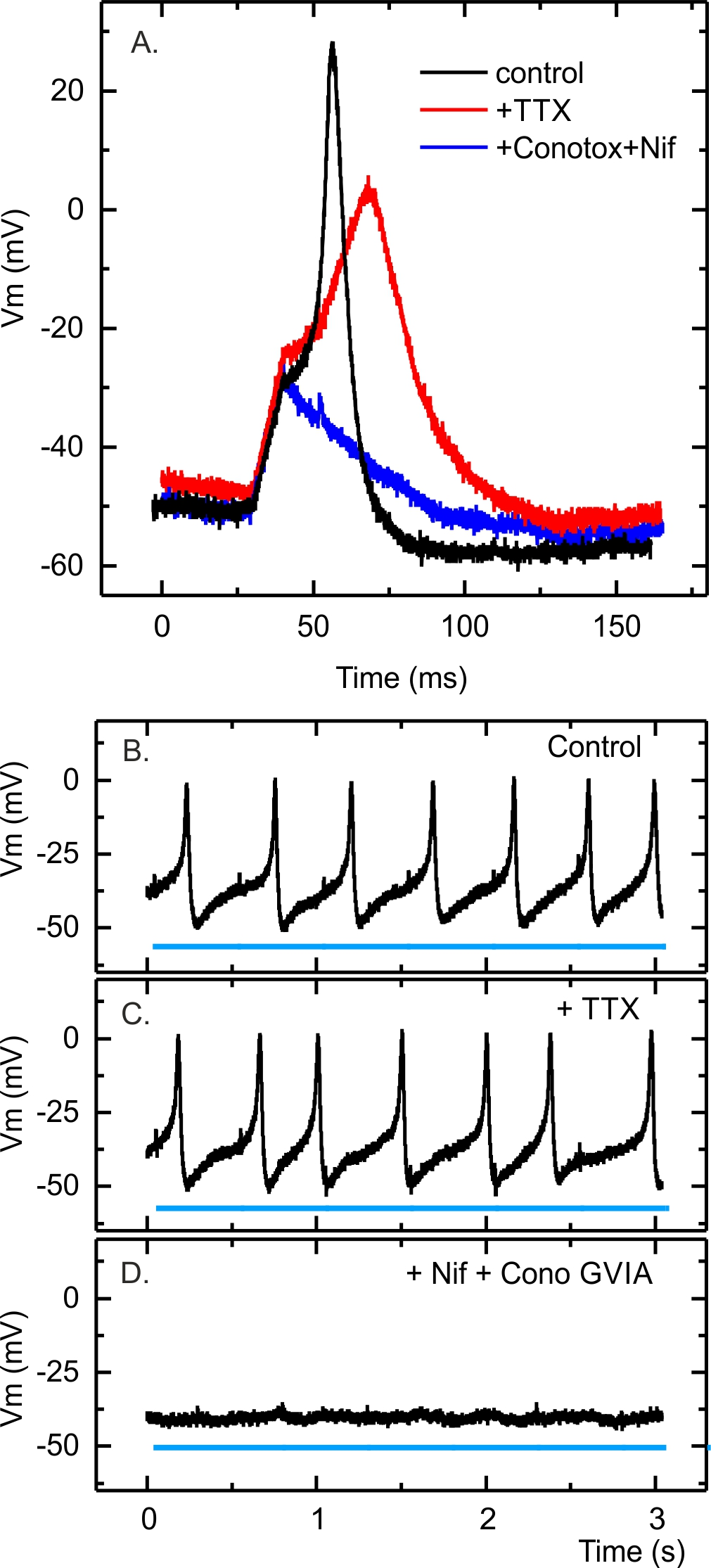


Figure S3. Pharmacological characterisation of light stimulated action potentials in GLUTag-S cells

A. Superimposed electrical activity in response to current pulses of 10 ms duration and 14-16 pA amplitude in a GLUTag-S cell in whole-cell current clamp in the absence and presence of ion-channel inhibitors. While APs were evoked in 0.1 mM glucose (black line) or the additional presence of TTX (3 μM, red line), further addition of nifedipine (5 μM) + ω-conotoxin-GVIA (1 μM)(blue line) prevented AP-firing (even after injections of bigger currents {not shown)). B. Electrical activity recorded in a light stimulated (blue line) GLUTag-S cell in perforated patch whole-cell current clamp in the presence of A. 0.1mM glucose, B. + TTX (3µM), C. +TTX (3 μM) +nifedipine (5µM ) + ω-conotoxin GVIA (1µM).


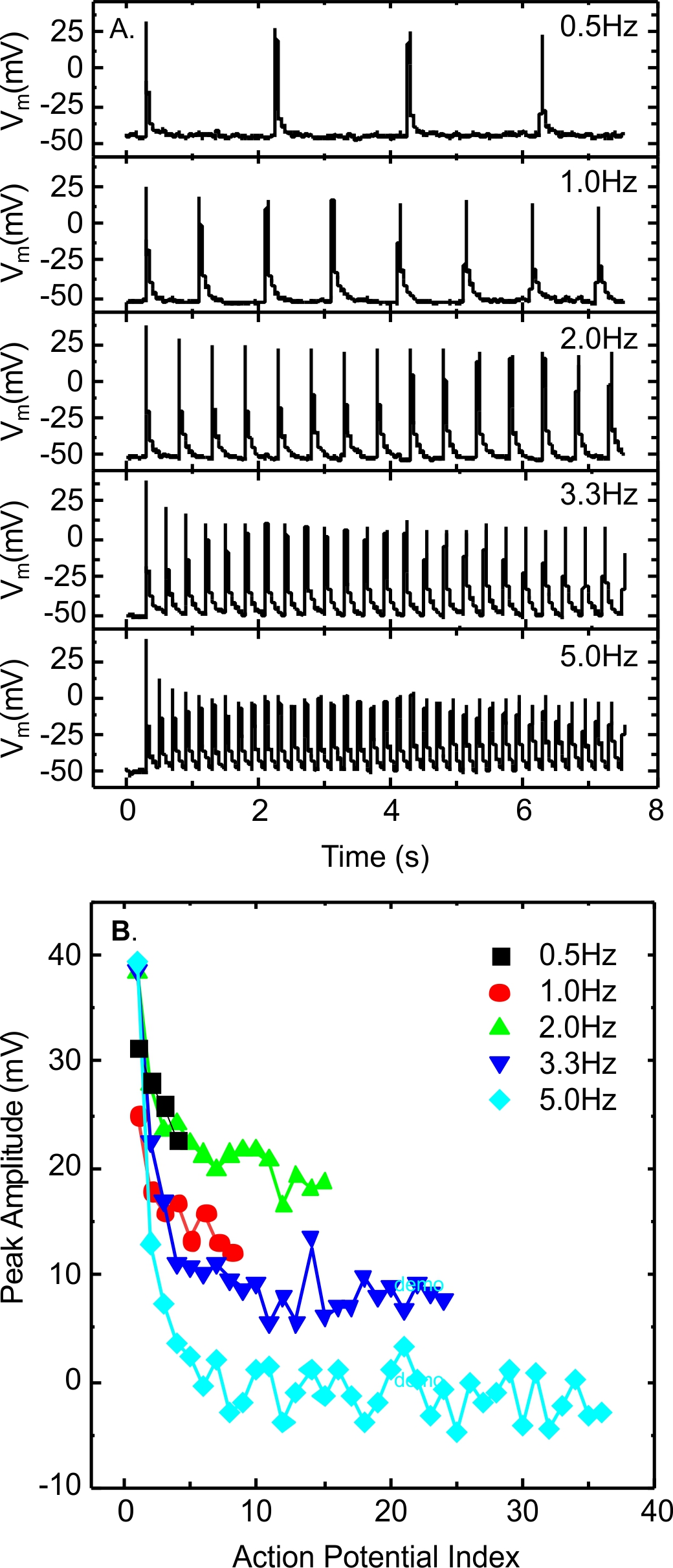


Figure S4: Light evoked action potentials in CheTA-transfected GLUTag cells in response to light pulses applied at increased frequency. A. Current clamp recording of a transiently CheTA-transfected GLUTag cell in conventional whole-cell patch clamp stimulated with light pulses of 10 ms duration applied at different frequencies as indicated. B. Peak amplitude of the evoked action potentials according to order in the pulse train. Note that the cell follows the imposed frequency, but that very little time is spent at potentials above 0 mV at higher frequencies.
